# Chloroform or Ether?

**Published:** 1896-06

**Authors:** John Freeman

**Affiliations:** Anæsthetist to the Bristol General Hospital


### CHLOROFORM OR ETHER?

BY

JOHN FREEMAN, F.R.C.S. Ed.,

*Anæsthetist to the Bristol General Hospital.*

---

THE question of which is the best and safest general anæsthetic continues to be constantly brought under the notice of the profession by the frequent occurrence of deaths during anæsthesia. The fact that opinions are still so divided shows that a good deal can be said in favour of both chloroform and ether; but one thing stands out pre-eminently, and that is, that when deaths do occur they are nearly always when chloroform has been used. It is also fairly evident that, so far, experiments on animals have not helped us much in coming to a right conclusion as to the safest anæsthetic to use for the human subject.

*Chloroform.*—One of the great advantages of this agent, and I believe reasons, speaking generally, why so largely used, is the very simple way in which it can be given even by one who has had very little experience in anæthetics. It is also fairly pleasant for the patient to inhale, and in the great majority of cases it answers the purpose admirably; but these advantages are counteracted by the treacherous way it acts in some instances.

A few of the deaths reported lately are good illustrations of the different ways in which chloroform appears to kill. Some of the patients were obviously not fully under the anæsthetic, as they were said to have "struggled, evidently feeling the pain," and immediately afterwards the heart and respiration stopped. Others in going under struggled violently and suddenly died. Another died just after he had been lifted from one place to another; while another succumbed apparently because the chloroform was administered to him whilst he was sitting upright.

It is not my intention to attempt to criticise the method in which the anæsthetic was given in these cases, because I think the whole circumstances should be taken into account. For instance, the nature of the operation may demand that the patient's head be raised; or it may be quite necessary to lift him from one place to another. There is one point I should like to touch upon in connection with these cases, and it is, that in those cases which died after struggling, it is almost invariably suggested that they died from an overdose of chloroform brought about by the inhalation of a large quantity of the anæsthetic in the deep inspirations that occurred between and after the acts of struggling. To say this in any case is to throw considerable blame upon the anæsthetist, as it means that he gave the chloroform in an unscientific if not a reckless manner. That it is possible for a patient to take an overdose in this way no one is likely to deny; but that it is the probable cause of death is, I think, very doubtful. These deaths from struggling have happened in the hands of experienced anæsthetists who must have been well aware of the fact that any rash pushing of the drug at this stage was particularly dangerous, and there is very little doubt they took every care that no chance of overdose was given.

There is another way of explaining these cases. When a patient struggles he always holds his breath, and it is generally understood that any obstruction to breathing, whether the chloroform is being given at that particular moment or not, is very likely to impede the heart in its action. If the breath is held for any length of time the pulmonary circulation and the right side of the heart become engorged, and in this way the heart's action is interfered with. The heart thus working under difficulties, now has another strain thrown upon it by the violent exertions of the struggling patient. It has been noticed many times that the heart will stand very little extra work in some patients during the inhalation of chloroform. The mere lifting of the patient from place to place, or sitting him upright, have been sufficient to cause death even when the respiration had been apparently good; but in these cases the respiration is embarrassed and the heart is already weakened by the over-distended condition of its right side.

Why then, since we have two well-recognised dangerous conditions present, each of which has in several cases produced death, must we bring in another cause, viz. the overdose theory, before we can satisfactorily explain these accidents? This overdose theory has far-reaching results, and it is quite possible that it is accountable for some of the deaths that occur in other ways. One not unfrequently hears of half an hour or more being taken to get a patient under with chloroform. All statistics show that the going-under period is one of the most dangerous; anyone, therefore, who unduly prolongs this period must subject his patient to unnecessary danger, and this is brought about by the fear of giving an overdose. The same sort of idea is shown again in those cases which die apparently from syncope, through the operation having been commenced before the patient was sufficiently under. Hence, we read of the patient "moving, evidently feeling the pain," or again, "the corneal reflex was present." All such cases as these seem to show that the administrators of the chloroform wanted to prove that the patients did not have an overdose. Dr. Hewitt, in his book on anæsthetics, says "there is about as much risk from administering too little as from administering too much chloroform," and the reports of these cases appear to point to the correctness of his view. Again, if most of the deaths under chloroform are due to overdose, as some seem to think, how is it that accidents happened with those who used inhalers that measured the chloroform vapour given to the patient? and why is it that most of the fatalities did not occur in the practice of those who make a rule of using large quantities of chloroform in their administrations? For instance, one celebrated anæsthetist thinks nothing of using as much as two ounces for a small operation which can only last a few minutes. Have we to go to such as these to find the majority of fatalities? It is more common to read of death taking place after only a small quantity of chloroform has been used. One case was lately recorded where the patient died from half a drachm. In two cases it was noticed that the heart stopped before the respirations, and several others read as though death was due to primary cardiac syncope. Chloroform, in some patients at any rate, appears to put the heart into a condition of instability—that is, in this state its action is affected and may be stopped by circumstances which at no other time have such influence on it. Hence, interfered respiration, the act of vomiting, the feeling of pain, &c., have all in their turn brought the heart to a standstill.

The large number of deaths that have happened lately in children shows that chloroform is not such a safe anæsthetic for them as was once thought.

Since deaths are so frequent under chloroform, the question ought to be considered whether we are quite justified in employing this agent. It is true the chance of an accident happening in any particular administration is very small; but there is no doubt that in all those cases that ended fatally the anæsthetists thought the same thing, and probably some of them told the patients as much. There are some who, because they have given chloroform a great many times without any accident, have come to look upon it as a safe agent; but whilst they have had good luck, others quite as experienced have not been so fortunate: besides, as only a small proportion of patients die in this way, many anæsthetists will chance to have a large experience without a death. But at the same time, since we have no means of telling which patient will take chloroform well, and which one will die from it—a strong, healthy man being just as likely to fail as a weak one,—it is doubtful whether we are doing the best for the patient when we proceed to give this anæsthetic, unless there is some special reason why it should be the one selected.

*Ether.*—This anæsthetic appears to be becoming more generally employed every year. There are several reasons why it is not used more. It requires an apparatus for its administration. Some might say this ought not to be a reason; but still, one may be called suddenly to give an anæsthetic where there is no ether apparatus, so chloroform has to be resorted to. Another reason is, that ether is a little difficult to give. While one who has never administered an anæsthetic before will be able to get a patient under fairly easily with chloroform, this is not usually the case with ether. Great difficulties may be met with by the beginner, and with a strong patient there would very likely be failure. Ether is not pleasant for the patient to take, and it is said not to relax muscles sufficiently in some cases. The most important objection, however, and the one which chloroform advocates use against it, is in its after-effects, particularly in regard to affections of the air-passages. These points about ether are worth some consideration. The difficulties connected with its administration are to a great extent preventable: it can be given in such a way that thirty or forty cases in succession will take it without there being struggling or any other difficulty; and when one sees a very muscular man go under without moving so much as a finger, as is frequently the case, it is difficult to believe that he is experiencing any very great discomfort. The causes of struggling, &c., are sometimes fairly obvious and may be due to too strong a vapour being presented to the patient at the commencement of the administration. The patient finding it impossible to breathe this, although he may try his utmost, naturally begins to struggle for breath. Another cause is, that the ether is sometimes commenced when the bag is only half full of air, and some that is in it will very likely be allowed to escape, through the face-piece not being applied properly, so in a minute or so the bag is empty. The patient tries to take an inspiration, when the bag collapses, as there was so little in it. Under such circumstances, is it surprising that the patient, finding he cannot get any air to breathe, should struggle? Again, giving too much fresh air at an early stage of the administration is a frequent cause of struggling. You may see a patient begin to take ether perfectly, and he may have got to the stage where consciousness is just being lost, the breathing being rapid and forcibly expanding the air-bag at each expiration. Should at this period the anæsthetist unguardedly allow two or three breaths of fresh air, trouble may be expected. A small quantity of fresh air at this stage will restore the patient's consciousness, and bring back sensitiveness to his air-passages. On the inhaler being re-applied, the patient instantly holds his breath, he feels and realises the pungency of the now somewhat strong ether vapour, and struggling, vomiting, and other troubles rapidly follow each other.

Such points as these make all the difference to the sensation experienced by the patient, and very little practice will prevent those who are learning to give anæsthetics from making such mistakes. This is why I hold that students should have the opportunity given them of learning to administer ether. When they get into practice they can please themselves whether they precede the ether with nitrous oxide, or use a little A.C.E. mixture until the patient is becoming unconscious. These are only details: the important thing is, that they will feel capable of administering properly what in future will most likely be considered the safest anæsthetic. I do not agree with those who say that ether will not sufficiently relax muscles for some operations. I believe that continued rigidity of the muscular system depends much more upon how the ether is administered than upon any peculiar idiosyncrasy of the patient, and I never meet with cases in which I cannot for all practical purposes completely relax the muscles. All patients, in going under with ether, pass through a stage in which there is more or less rigidity. This passes off in most patients in a minute or two, but there are two classes of individuals—the alcoholic, and the very muscular—in which this may not happen. These remain rigid for some time; and you may have the ether on full, and limit the supply of fresh air to a large extent, and yet the spasm continues. These are exceptional cases and they require different handling, but it is a mistake to think that nothing more can be done to get them under. The appearance of the patient in this condition gives one the key to the difficulty. The muscular spasm is a general one, and so the muscles of respiration are included. The result of this is, that the patient becomes deeply cyanosed. On giving a plentiful supply of fresh air to remove this cyanosis, one notices at the same time that the muscles begin to lose their rigidity; so in these cases, when I find, after a good trial of ether, that the muscles do not relax, I remove the inhaler altogether, and let the patient have fresh air until his normal colour returns and the rigidity begins to subside. Of course, this procedure brings the patient half round from his anæsthesia, and his reflexes become active again, so on re-applying the inhaler it is very necessary to begin with a weak vapour; a strong one, by causing holding of breath, &c., would bring back all the rigidity in a very short time. I have not met with a case yet which failed to go under completely on this second attempt, but necessarily the time taken to get one of these patients under is much longer than in an ordinary case.

With regard to the after-effects of ether, there are only two worth considering. The first is vomiting. In comparing the vomiting that takes place after ether with that of chloroform, so far as I have been able to observe, there is very little difference between the two. More patients vomit after ether than after chloroform, but the ether vomiting generally passes off more quickly. That long-continued vomiting, going on into the second or third day, which now and then follows chloroform is very rare after ether.

Now we come to the chief objection to ether as an anæsthetic; viz. that it sometimes produces affections of the air-passages: were it not for the possibility that ether may indirectly cause death in this way, chloroform would have been doomed long ago. Unfortunately, it is impossible to speak definitely about this point, because there may be other causes to produce these troubles at the time of an operation besides the ether. I have made enquiries on this subject from those of experience, and the general opinion is that chest affections resulting from ether are extremely rare. Dr. Hewitt, in dealing with this subject, says "there has undoubtedly been gross exaggeration." My own experience is a little interesting. In 1600 administrations of ether to patients of all ages, from 6 weeks up to 80 years, (many of them, too, were in long operations lasting two or three hours), I have met with one patient who had some bronchitis after. This was a woman aged 23, who, however, made a good recovery. The anæsthetic received all the blame; but I have also had a case in which bronchitis followed an operation in which chloroform was the anæsthetic. In this case, however, the bronchitis was looked upon as a sort of coincidence, or perhaps due to the exposure of the patient during the operation (one can make a very good guess as to what would have been thought if ether had been used in this instance). One other case I have met with gives a good illustration of how easy it is to blame ether for what it does not deserve. It was a child six years old. Its nurse had not noticed anything the matter with it, and there was nothing definitely wrong with the chest. I used A.C.E. mixture, and in going under the child coughed several times which made the operator remark "the ether was making the child cough a good deal." For some days afterwards the cough was very troublesome, and was still supposed to be due to the "irritation of the ether;" but on the sixth day after the operation the child developed a characteristic "whoop" with its cough, and after this it went through the ordinary course of whooping cough. Ether has been given at this hospital, by others, many hundred times without there being any other case in which it was suspected to have caused any chest trouble.

From such experience as I have had of these two anæsthetics, I cannot help thinking that a patient is in a much safer condition under ether than under chloroform. With ether, so long as the patient is kept sufficiently under to prevent holding of breath, vomiting, etc., there is no trouble, and one can depend upon having a good warning if the patient is not quite satisfactory. Up to the present time, no patient under ether has ever given me the least anxiety. I cannot say this of chloroform. Sometimes with a cause such as putting a gag into the mouth, or placing the strap of Clover's crutch round the back of the neck, and sometimes without any obvious reason, I have had patients who were in a condition of considerable danger. Because of this, I now make a rule of always using ether, unless there is some reason for its contraindication.

Sir B. W. Richardson, in a recent article on chloroform deaths, writes: "There are certain persons, say about 1 in 3000, who at all times are ready to die." He has designated them "the morituri," and says they are the common victims of chloroform. If this is the real explanation of these deaths, it is one more reason why ether should be used, because whilst chloroform is constantly finding out these cases, ether scarcely ever does so. If, as statistics often show, in 10,000 administrations of chloroform four such deaths occur, and in the same number of ether cases no such accident happens, there must be some very important difference between these two drugs.

I think we have not to look far to find the reason of the greater safety of ether. The full bounding pulse of ether anæsthesia shows how much the circulatory system is stimulated by it, and the rapid and deep character of the breathing proves the same influence on the respiratory organs. So if instead of using an anæsthetic which has a tendency to depress both the respiration and the circulation (as chloroform has), we employ one that has a directly stimulating effect, we are much more likely to tide these "morituri" over their operations.

---

### DERMOID CYST OF A BRANCHIAL CLEFT.

